# Concussion of the Brain

**Published:** 1844-10-19

**Authors:** 


CONCUSSION OF THE BRAIN. FRACTURE OF THE BASE OF THE SKULL.

William Lawrence, aged sixty-four, was admitted into Westminster Hospital, under Mr. Hale Thomson, at 3, P. M., of the 20th of May, having fallen from a scaffold, about fifteen feet in height, upon his head.

On examination, the scalp was found considerably contused over the occipital bone of the left side, pupils contracted, considerable bleeding from the left ear; he was very irritable and had the appearance of a man half drunk. Soon after being placed in bed he became gradually insensible, pupils were dilated, breathing stertorous, mouth drawn to the right side. He had also slight convulsions. At six, P. M., the pulse having risen and become full, he was bled to fourteen ounces, without any relief to the symptoms. At eight o'clock he was again bled to sixteen ounces, after which the breathing became quieter, and the pulse less full, but the pupils remained dilated, and he had several convulsive attacks during the night, but not so severe as before the last bleeding.

Early in the morning of the 21st a turpentine injection was administered, which acted freely on the bowels; pupils still widely dilated, breathing quieter. The enema was repeated daily till the 28th, and nothing given him but tea. On the 28th, on rousing him, he complained, for the first time, of pain in his head; pupils still dilated. Ordered to take two grains of calomel every three hours, for four doses, and then every six hours for four more, and afterwards to take it night and morning only. To have a pint of milk daily, with tea. He continued the calomel until the 2nd of June, when the mouth and gums being very sore, it was left off. The pupils are less dilated and act by the stimulus of light. He is much more sensible; pain in the head less; bowels rather confined. Ordered a dose of aperient medicine.

4. Better. To continue the milk and tea.

6. Pupils still dilated; complains very much of his mouth; bowels regular; pulse full.

10. Is now quite sensible, but is rather deaf; pupils dilated, and act but slightly; tongue when protruded is drawn a little to the left side, and is thrust out by successive efforts. A pint of milk daily.

15. Going on well; complains of being very hungry.

20. Is now able to sit up during the day; the pupils remain dilated, and there is slight hesitation in his speech; pulse full and hard; bowels open.

From this date he continued to improve gradually and steadily. He had occasional attacks of irritability, as was evinced by unusual garrulity, but he was so far recovered by the middle of July, as to be able to leave the hospital, having been previously admonished to avoid all kinds of stimuli.—*London Lancet.*

